# Light to the Gentiles

**Published:** 1889-12

**Authors:** W. S. Elliott


### LIGHT TO THE GENTILES.[1]

BY W. S. ELLIOTT, M.D., D.D.S., M.D.S.

ONE-THIRD of a century of active devotion to our work ought surely to have evolved something worthy of record; and so it has, perhaps; but it is difficult to concentrate the nebulæ of diffusive experience into anything like a planet of tangible proportions. Each day's work is suggestive; and it is thereby that we move forward, forcing the light of accruing knowledge into the void of darkness, of ignorance, and inexperience. Moving, we would

> "Gather up the sunshine bars
> That fall around like jewelled stars."

This congregated mass of intelligence is but the effulgence of the noon-day, limiting the shadows of interference to a minimum degree of extension; and that the gentile mind may not continually repose within the shade of indifferent attainment is this Society's mission.

Dentistry is said to be a science of demonstration, while medicine is more or less empirical. But the science that leads to success in practice in either department is rational in its working, and not the outcome of mere chance effort.

In giving consideration to this subject, opinions must give way to proofs, and conclusions follow only on substantial scientific premises. Pure assumption is the dark lantern that fails to enlighten,—scientific penetration alone opens the understanding to a full recognition of true genetic evolution. In studying the successive steps of progression, we would postulate a Power which is parent of the forces, and which becomes the instrument or medium of consummation of purpose. Step by step, as sequence and consequence, should observation be governed. It is thus that metamorphoses are distinguished and knowledge gained. Some phases of accepted knowledge will prove only inferential; but when inference in any given case has developed to positive assurance, under the usual rendering of the law, then may we safely dwell upon the undefined

[1] Read before the New Jersey State Dental Society, at Asbury Park, July 18, 1889.

in the series as true to satisfaction. There are apparently missing links in the chain of development in every process of natural law; and it is safe to assume that the gaps will in due time be filled by tangible realities as well as by the possibilities of inference.

Knowing the law, we must follow its tendencies, despite seeming variability; thus may the energies of aggressive inquiry be conserved to timely and effective results. This is the incentive to persistency in every line of research, and the reward is as inevitable as the law is true to its fulfilment.

Diagnostic skill depends upon an habitual observation of the deflections from normal processes, and the latter can be fully comprehended only by the following of the serial steps of "togetherness" under the rule of typical presentment. Change, which is the "immortal delight of creation," and which is the good angel of our being, resides primarily in the ultimate atom. Here is stored the energies of developmental entity, which, through differentiated environment, are set free to the accomplishment of purpose. How important is it, then, to know the value of endowment bestowed upon these, and the possibilities of which they are possessed. A perfect environment and a perfect conformity to that environment is the consummation of perfect being.

We build, sometimes, better than we know; and past efforts have shown how successfully we battled with microbes by the use of the older antiseptic agents, when the enemy was unseen and unestimated. The rule of aseptic treatment, now so generally adopted, is the outcome of scientific research and provings; and all former attempts in this direction become the more rational as we recognize the possibilities of contagion.

To speak now more especially of features of actual practice, you will bear with me if I should be diffusive in my references.

First, of the antiseptic agents of later discovery. In the hydrocarbon group of chemical compounds do we find the most efficient ones, and of these, my choice, under most conditions, is the *camphophenique* of the Phenique Chemical Company, of St. Louis. It is antiseptic in a high degree, and meets most satisfactorily the large majority of cases of daily practice. It is non-irritant, and not unpleasant to taste or smell. It is set forth as a definite chemical compound, having for its formula $C_8H_{11}O$. But this, I think, is not strictly true. My impression is that it is a mixture of two distinct molecules of camphor and carbolic acid. The formulæ will bear me out in this assertion.

Carbolic acid is represented by $C_6H_5(OH)$. Camphor,—$C_{10}H_{16}O$.

The chemist has merely added these numbers together and reduced the product to its lowest denomination, thus:

$$\begin{array}{r} C_6H_5(OH) \\ C_{10}H_{16}O \\ \hline 2)C_{16}H_{22}O_2 \\ \hline C_8H_{11}O \end{array}$$

No interchange of atoms can take place until there is a stronger affinity set up by the presence of other atoms of other broken molecules while in their nascent state. It will not be claimed, perhaps, by the manufacturers that the formula as given is anything more than an empirical one, since the exact changes, which take place when certain crystalloid substances of the camphor group are brought into contact with the halogen derivatives, are not well understood, although their mutual liquefaction has long been known. The field is new and comparatively uninvestigated.

But I will not detain you by these technicalities, though the study of them is interesting, and of more importance than some I know of are willing to concede.

At a late meeting of the Massachusetts Dental Society, a paper was read by a Southern gentleman, extolling sterilized sponge as a dressing for exposed pulps. He cited numerous instances of complete success during a period of two or three years. The showing was of such a nature as demanded a philosophical statement of the process of repair, and a request was made for an exposition of the reasons why sponge was selected in preference to other materials which had heretofore been used for the purpose. No satisfactory reply was made, and only a general statement that the sponge prevented mechanical pressure upon the pulp when the cavity was filled; and that it induced a deposit of secondary dentine at the point of exposure. It will be profitable, perhaps, if we inquire into this subject a little, and ask if such practice is in harmony with the law of repair and reproduction. Sterilized sponge has been used in the treatment of lesions of the soft tissues with considerable success; and it was a curious phenomenon that the sponge thus used was found to have been taken up by integration and to have become a part of the new growth. It is evident enough that the porosity of the material favored the retention of a protoplasmic mass, which is held in a kindly way to encourage propagation. Its disappearance called for an explanation, which I have never seen published; nor would I presume to assert the whole truth in what I would advance, but merely infer that the chemical elements of the sponge were of a nature quite in harmony with physiological requirements and such as presented pabulum suitable for nourishment of the tissues. The components of sponge are gelatine, albumen, carbonate of lime, chloride of soda, magnesia, silica, iron, sulphur, phosphorus, and traces of iodine and bromine. All these substances stand in favorable relation to the constituents of the blood, and, through the chemico-vital energy, are appropriated the same as if presented to the parts through the blood circulation.

Now, returning to the treatment of the dental pulp, it does not seem probable that in this the sponge would play the same *rôle*, since it could not become saturated with any effusion from the pulp that would be organizable. At best, there would be only a slight moisture, and the sponge could take no part in the process of repair. It is the function of the globular bodies to lay down the lime salts, and the territory of repair is within the walls of the pulp-cavity rather than in the cavity of decay, conditions unlike the flesh lesions just mentioned.

If an exposed pulp is conserved by such a dressing, it is due, more likely, to aseptic conditions rather than to the sponge *per se*.

I have referred incidentally to the waste of energy incurred through the want of the knowledge of basal principles, and how such energy can be economized by a direct application of those principles when understood.

Therapeutics become the means to the end when combating disease, and in the department of mechanics every appliance which will help to direct this energy to the purpose sought should be available; and perhaps the most appreciable instrument of modern practice is the dental engine. Here I desire to make a point which, it seems to me, has been grossly overlooked, and you will excuse me for the reference. Seventeen years ago I had the pleasure of introducing to the profession an engine known as the Suspension. To-day, I am able to declare—as proven by those who have had it in constant use since that time—that it fully sustains the claims then made for it. I apprehend that its merits are not fully appreciated, for the reason that it has not been presented to the profession with as much push as has other inventions. With an independent motive-power—water or electricity—it becomes the *ne plus ultra* of all implements. There is in it a vast latitude and freedom of motion; no back lash; no slack bands; nothing to encumber floor-room; no necessity for a great amount of machinery to adjust and keep in repair. In referring to this instrument I have no interest, other than to remind you of the best of all.

Again, allow me to touch upon an item which you may think of but little moment, yet which embraces problems of interest and importance, when considered in a general way. I refer to the "setting" or consolidation of amalgam. I have asked frequently for the reasons why amalgam, freshly reduced from the ingot, should "set" quicker than when the filings were a few weeks or months old. You will admit the premises. The only answer obtained is that oxidation prevents quick setting; the grains of metal become oxidized through exposure, and crystallization is thereby checked. This is not satisfactory, since washing the filings free from the oxide does not favor the process of hardening. To my mind, the true answer is this: The friction of the file or cutter imparts to the granules a magnetic polarity which they are capable of holding for a limited time, but which energy is dissipated after a few weeks. Crystallization is an exhibition of kinetic force in perfect correlation with the stasis of magnetism, and the process is quickened by the transfer of the stored energy of the one mode to that of the other. Crystallization is checked in proportion as the magnetic energy is dissipated.

I do not bring these items forward as of particular importance in themselves, only as instances of opportunity that occur in practice when valuable information may be obtained by a habit or disposition to know the inwardness of things seen; and without holding you longer, you will accept the offer of my desire to urge directness of procedure in chemistry, therapeutics, and mechanics by as complete an understanding of each as may be obtained through a study of the laws which pervade and control all the operations of nature.

It is thus that the light of knowledge will penetrate the dark places and the subtile mysteries of being made to develop into substantial correspondence with observed phenomena.

